# Letter from Dr. C. T. Cushman, to the Baltimore Editor

**Published:** 1850-01

**Authors:** C. T. Cushman


THE AMERICAN

### Journal of Dental Science.

Vol. X.] JANUARY, 1850. [No. 2.

### ARTICLE I.

*Letter from* Dr. C. T. Cushman, *to the Baltimore Editor.*

Columbus, Ga., *Nov. 7th*, 1849.

Dr. Harris:

*Dear Sir:*—In examining some old volumes of medical journals, in a friend's library, I came across an interesting paper by the late Dr. James Gardette, dentist, of Philadelphia—the same alluded to by his biographer, Dr. E. B. Gardette, in your Dictionary of Dental Science, art. Gardette. The subjects of it are, the *Transplantation of Teeth* and the *Luxation of Teeth*, illustrated by reports of cases; and I regard it as extremely valuable, in affording the enlightened views and vast experience of so distinguished a *father of dentistry*, as was, emphatically, Dr. Gardette. I therefore felt a strong desire to have it preserved in my own library, as well as that the information it contains should be disseminated among the profession of the present day; for I do not know of any other on record, within their reach, that contains similar reports, showing the *practical results* of the methods discussed.

*Mr. Hunter* gives no case of transplanted teeth, excepting one operation, performed with a view to arrest a diseased action in the *alveolus*—which he regarded as successful;* and another, showing their liability to caries.†

The serious and *fatal* cases, cited by Dr. Gardette, incontestibly prove the error of Mr. Hunter's preconceived opinion, that disease could not be so communicated from one person to another:—"not that I believe it possible to transplant an infection of any kind from the circulating juices."—*Hunter*.‡ His work, likewise, gives but one case of replacing teeth which had been luxated, and those by accidental violence. They were reinstated "some hours afterwards"—"after a month they were as fast as any teeth in the head;" and had so remained four years, when the case was reported.§

*Maury* gives two cases of transplantation, (his only ones,) in his own practice. In one, the tooth never got firm, and had to be removed in "eight or ten months." The other remained, always in a loose state, fourteen years, and was then just ready to drop out.‖

A fatal case of transplantation is quoted in *Fitch's* Dental Surgery, p. 413.

Besides the interest of the various cases detailed in Dr. Gardette's paper, it contains collateral information that may be new to many of us. It would seem that the author is justly entitled to the credit of having *first* proposed the operation of luxating a painful tooth, in order to sever its nervous connection, and then replacing it in its socket; as, according to his dates, he practiced it about *forty-seven years ago;* which is, per-

* N. Y. ed., p. 20, part ii. † P. 39, part ii. ‡ P. 36, part ii.

§ P. 37, part ii. ‖ Philad. ed., p. 176.

haps, a year or two farther back than the time it was proposed by *Mr. Fox*, who is generally regarded as the originator of that practice. However, it was not so successful in his hands as in Dr. Gardette's.

It is probable, too, I should infer, that the *first gold plate* for sustaining artificial teeth, made in America, was adapted by Dr. Gardette; the one he cites having been constructed by him *sixty-seven years ago*, "after the mode he had invented about that time."

It will be seen, further, that he gives the name of *Dubois Dechman* as the "first" inventor of porcelain teeth. The credit of this invention is awarded, by M. Audibran, to *Duchateau.* Mr. Fox names *M. Dubois-Chement* as the inventor—who, it is admitted, carried the art of their manufacture from France to England, but kept it always secret. According to Audibran, *M. Dubois Foucon* is the *first who made it public.** I have never before seen the name of Dechman in this connection.

I will conclude, sir, by expressing my earnest hope, that you will renew this paper—a transcript of which I herewith send you—by giving it a deserving place among the valuable literature of our profession—in the *American Journal and Library of Dental Science.*

With sentiments of respect,

C. T. CUSHMAN.

* Traité sur les Dents Artificielles, p. 45.

[From the "Medical Recorder"—Philadelphia, 1827.]

*Observations on the Transplantation of Teeth, which tend to show the Impossibility of the Success of that Operation: supported by a New Theory.* By JAMES GARDETTE, Dentist.

I HAD, a considerable time since, determined to write on the subject of the transplantation of teeth, from the mouth of a living person into that of another; but my great occupations, and the diffidence I always had in writing English for publication, have prevented me from attempting it; and it is probable I should never have done it, had I not been encouraged by my friend, Dr. Mease, who, after a conversation on that subject, lent me the first volume of the Memoirs of the Medical Society of London, in which several cases of disease succeeding the transplantation of teeth are published, by J. C. Lettsom, M. D., &c., one of which happened in August, 1785. I read the articles with great attention, and found I was acquainted with the first case, which had been related to me by the gentleman, J. Y., now a citizen of Philadelphia, in whom it had occurred a few years before.

The reading of those articles, and the observations of my friend, Dr. Mease, who is very much alive to the dissemination of useful knowledge, determined me to write this paper, and at the same time to endeavor to prove the impossibility of transplanting teeth from one mouth into another, with the success expected and promised by the operator, viz. that the tooth transplanted will remain firm, and as useful as the tooth which had originally grown in the jaw.

I shall preface this paper, by informing my readers that I arrived in Philadelphia in June, 1784, and began to practice my profession; and that Mr. Lemayeur, with the reputation of an eminent dentist, had transplanted one hundred and seventy teeth in this city, in the course of the winter of the years 1785 and 1786, as he told me himself, at Baltimore, in the fall of the last mentioned year; and that, of all those transplanted teeth, not one succeeded! Some became firm, and lasted, more or less so, for one or two years, in the sockets in which they had been inserted; but those cases were very rare. In the course of my practice, after that time, I had occasion to extract at least fifty of these transplanted teeth—most of them without an instrument, with my fingers only—and to replace them by artificial teeth. Many accidents occurred to the transplanted teeth, while they were growing firm, and some never got firmly fixed in the sockets at all. I shall now relate some cases of that nature, which happened to teeth transplanted by Mr. Lemayeur, which, I dare say, will be recollected by some persons now living in this city, and perhaps by relations of the persons who were operated on at the time.

Mrs. A. W., a lady of great respectability, had several, I believe *three*, front incisors of the upper jaw transplanted; after suffering for a considerable time, the transplanted teeth not becoming firm, she was obliged to have them extracted, and artificial teeth replaced in their room.

Miss W., a young lady at a boarding school, had the four upper front incisors attempted to be transplanted, but they never became firm; the gums were so inflamed and ulcerated, that the disease was communicated to the lip, so as to form a complete adhesion with it; they

6*

were separated by scarification, but the adhesion of the gum and lip could not be prevented, until the transplanted teeth were extracted; which being done, the lip and gum perfectly cicatrized in a short time; the space was then filled with artificial teeth.

I was informed that, about the same time, a young lady of New York, Miss S., had a large front incisor transplanted, in the upper jaw, which produced a disease, judged by the physicians who attended her, to be the *lues venerea.* This young lady was so affected by the disease, that, notwithstanding all the medical aid given her, her health declined, and, after considerable suffering of mind and body, she died.

I was also informed, that a Mr. T., of Virginia, had a front upper incisor transplanted about the year 1790, by the same dentist, the exact time not being remembered, which occasioned much inflammation in the gums and eyes. After some time, the *opthalmia* became severe, and other symptoms justified the opinion that the *lues venerea* had been introduced into the system by the transplanted tooth, which, no doubt, was taken from an unsound subject. I was informed at the time, that Mr. T. had lost his sight, and that after lingering for some considerable time, he died.

Mr. W. H., of Philadelphia, had three upper front incisors transplanted in London, in the year 1784 or 1785, under the superintendence of the distinguished surgeon, John Hunter; the operation was performed with all the care and skill possible, and the teeth became firm in a short time, without any accident of importance. I saw the gentleman in this city about five years after the transplantation of the teeth, which, at that time, were somewhat loose. He consulted me as to the cause of the looseness of the transplanted teeth. On examining his mouth, I found that the teeth of the under jaw, directly under the transplanted ones, struck against them on their internal surface; and I judged that the continued shock occasioned by the under teeth, was the real cause of their looseness; but that the original cause of the under teeth touching the upper, was the inflammation of the gums of the transplanted teeth, which caused their dropping down, and thus to meet the under incisors opposite to them. In order to remedy this inconvenience, I proposed to shorten the under teeth, which I did with a file; I then advised the gentleman to make use of an astringent wash to brace the gums of the transplanted teeth, which were inflamed, and somewhat spongy; my prescription was followed, and the teeth became firm in the course of two weeks. I did not see the gentleman's mouth after this for a considerable time, as he lived out of the city. But having had occasion to see him some years after the time I attended him, I perceived he was without the transplanted teeth, which he had never replaced.

Of all the transplanted teeth that I ever saw, or heard of, none have lasted so long as those transplanted in the mouth of Mr. W. H.; for they remained very firm for about five or six years, and lasted about as long in a loose state, which increased until the teeth either dropped out, or the gentleman extracted them himself with his own fingers; for I am persuaded they were not extracted by a dentist.

None of the teeth transplanted by Mr. Lemayeur, in Philadelphia, remained firm two years; and in two or three cases which I have seen, of teeth transplanted by other dentists, they did not remain firm one year.

Mrs. J. P. had a large incisor of the upper jaw transplanted in London, also under the care of the celebrated John Hunter, in the year 1780, or thereabout, (the time which was mentioned to me not being remembered,) the tooth became firmly fixed in a short time; but, about a year after its transplantation, a small discharge of matter was perceived issuing from the under edge of the gum, on the left side of the transplanted tooth; but this was not much regarded at the time, being very trifling. The daily attention paid the teeth, by washing and brushing them, prevented the lady from taking notice of its progress for some years.

Having determined to leave London and come to Philadelphia, after the peace of 1783, she had, a few days before her departure, her mouth examined by her dentist, who readily found that a *fistula* was the result of the continual issuing of a small quantity of pus from the socket of the transplanted tooth. It was then judged by him, that the tooth could not remain long in its place; he advised the lady to have a tooth prepared,* that could be easily fixed in the place of the transplanted tooth by a dentist, should there be one in Philadelphia, (which, it appears, was much doubted at the time in London.) After her arrival in this city, Mrs. P. consulted Dr. W. Shippen, who, after examining her mouth, determined that the transplanted tooth should be extracted. The doctor sent for, and asked me if that was not my opinion; after examining and probing that part of the socket which could be reached with a probe, I

* It was a porcelain tooth, made by Dubois Dechman, a French dentist, in London, who first invented the manner of making artificial teeth out of porcelain, and which has been so much improved since by several dentists, and particularly by *Fonzi*, an Italian dentist and chemist at Paris.

found that the left side of the root of the tooth, as also the socket, were completely decayed to the extremity of the root, which was perfectly adherent to the socket on the right side, the tooth being still very firmly fixed, notwithstanding the existing caries.

I told Dr. Shippen that the tooth ought to be extracted, in order to cure or dry up the *fistula.* But there was some difficulty in extracting the tooth without breaking that part of the *alveola* which was completely ossified with the right side of the root; and which I thought I could avoid, by means of an instrument which I would cause to be made by our old and only cutler, Mr. Schively, and which I described to the doctor, as follows, viz. the blade in the form of a narrow straight scalpel, thin, and very sharp-pointed.

After having informed the doctor of my intended manner of performing the operation, he approved it. At the time fixed by the lady, I operated in the presence of Dr. Shippen and a gentleman, a friend of the family, (Mr. John Mifflin,) in the following manner:

I separated the adherent plate of the socket from the root of the tooth, with my sharp-pointed instrument, with all possible care, in the space of about two minutes; I then removed the tooth with a straight forceps, with the greatest ease imaginable.

The exfoliation of that part of the socket which required it, and the cicatrizing of the gums, required nearly a month, when I replaced a natural tooth, mounted on a gold plate, after the mode which I had invented about that time; this tooth resembled so perfectly the large *incisor* which remained, that no person could perceive the difference.

The transplanted tooth being examined after extraction, it was found that one half of the root had been destroyed by caries, longitudinally to its extremity, which proved the absolute necessity of its removal.

It is possible that the dentist who transplanted the tooth, finding the root of that tooth too big, filed off some of its thickness, (as I have heard of that being done sometimes,) to let it go easily in the socket; the periosteum having been removed, the root could not adhere to the *alveola* on that side; and that may have occasioned the formation, and of course the emission, of the purulent discharge spoken of. In order to establish my theory, I shall cite some cases which have occurred to me in the course of my practice, which will prove that a diseased tooth, taken from its socket, the cavity in it plugged, and the tooth replaced in the same socket, will become, in the course of ten or fifteen days, as firmly and solidly fixed as it was before the extraction, and last for a great number of years, and sometimes during life. This is certainly a tooth transplanted, to all intents and purposes, but in the same socket from which it was extracted. If, therefore, another tooth could have been found, the root of which was exactly of the same length, size and form, it might have been placed in the socket of the tooth just spoken of, and it would certainly have become as firm, and have lasted as long as the tooth which had grown in that socket.

I have frequently partially extracted and returned to their sockets, small and large molars, which had been very painful, after having cut the gum on the side opposite to that on which I intended the tooth to fall, in partially extracting it. The purpose of this operation is to separate or rend the nerve asunder, so as to prevent the tooth from giving pain in future; the tooth is then put back into its socket, permitted to become firm, and the cavity is then to be plugged: this I always did with full success. It has sometimes happened that a dentist has extracted a sound tooth for a bad one, either by his neglect in ascertaining the tooth to be extracted, or by misinformation from the patient. If such tooth is replaced in its socket immediately after extraction, it will certainly become as firm and useful as ever.

All that has been said will prove, I hope, that a tooth taken out of its socket, and put back in its place, will become firm and useful; therefore, if a tooth taken from another subject, the root of which is of the same shape, length and size, is placed in the socket of the tooth extracted, it will certainly become as solidly fixed as the original one. But the dentist who transplants it must judge that the roots of both teeth are precisely alike in size and shape, before he sees either: that being impossible, the operation can, therefore, not succeed.

In the year 1801, I was requested to call on Miss B., a young lady of great respectability, who had suffered extremely from pain in a front tooth. I found it was the canine tooth of the left side of the upper jaw which caused the violent pain. I was requested to extract the tooth affected; but I observed to her, that the loss of that tooth would be very great, and that it might be preserved by replacing it in its socket after extraction, and that it would become as firm and as useful as ever. After explaining the manner in which I should perform the operation, she consented to have it done, and I proceeded in the following manner: I extracted the tooth with a straight forceps, cleared the cavity of its carious parts, filled it with gold, washed it in warm water, and inserted it in its *alveola;* all this was done in the space of five minutes. I then requested Miss B. to bite a piece of flat cork, (which I had prepared for the purpose,) several times in the course of the day, and to wash her mouth frequently with a slightly astringent liquid, which I prescribed; if I recollect right, the tooth was perfectly firm the twelfth day. That tooth rendered service to the lady for nearly eighteen years, as I extracted it, I believe, seven or eight years since, having become more carious, and therefore troublesome.

It is to be observed, that it is only the incisors and canine teeth which are attempted to be transplanted, as they have but one root. It is therefore the same species of teeth which I have extracted, plugged and replaced in their sockets; I have performed the same operation frequently in the course of the last twenty-five years of my practice, with the same success as in the case last mentioned. I will, however, detail the particulars of one case, in which the tooth extracted and replaced was a small molar of the under jaw. This operation was performed in the mouth of a lady of this city, Miss ——, and I expected complete success; but, on examining the tooth after extraction, I found that the extreme end of the root being bent, it broke in the extraction, and the piece so bent, (about an eighth of an inch,) remained at the bottom of the socket; notwithstanding the accident, I replaced the tooth in its socket, after having plugged it, in the hope that a callus might be formed, and a junction take place. The tooth was replaced for nearly a month before it became firm; but no kind of inconvenience was experienced, either by inflammation or pain. The tooth lasted in that situation full six years, when it became troublesome and a little loose. I extracted the tooth in the course of the seventh year; the broken piece of the root remained at the bottom of the socket, which healed completely, and has never given trouble from that day to this, although six or seven years have elapsed.

About eight and twenty years since, I was sent for by Miss G., a young Quaker lady from Virginia, who was in great pain from a large molaris of the upper jaw, and confined to her room. After examining the tooth complained of, I found it to be a very large one, with but a small cavity in the center of its crown. I proposed the operation of partially extracting the tooth, replacing it, and to plug it when it had become firm. The late Dr. Kuhn attended the young lady at the time. I informed him of my intention, and explained to him how I should proceed; the doctor having advised the young lady to let me make the experiment, the operation was performed. About two or three weeks after, I filled the cavity with gold, and I never heard any more complaint from it. I might cite several other cases of the same kind, which succeeded as well; but let these suffice.

My opinion, therefore, is, that teeth cannot be transplanted from one mouth into another, so as to answer the intended effect; that is, that the transplanted tooth will not become as firm and as useful as the one which it has replaced, and last as long till destroyed by caries or accident. The reasons which I give in support of this opinion, are those already advanced, that the root of the tooth which is to replace the defective one, should be of the same *length, size and shape* of the root of the one which is to be replaced, and that the dentist is obliged to judge of that without seeing either. I therefore believe that there are a thousand chances to one, against the success of the operation of transplanting teeth from one mouth into another, if not entirely impracticable.

